# The role of radiotherapy in the management of metastatic rectal cancer: A narrative review on the opportunities for non-operative management and organ preservation

**DOI:** 10.1016/j.ctro.2025.100976

**Published:** 2025-05-04

**Authors:** Reza Ghalehtaki, Parmida Sadat Pezeshki, Amirali Azimi, Fatemeh-sadat Tabatabaei, Nina N. Sanford, Krishan R. Jethwa

**Affiliations:** a Radiation Oncology Research Center, Cancer Research Institute, Tehran University of Medical Sciences, Tehran, Iran; b Department of Radiation Oncology, Cancer Institute, IKHC, Tehran University of Medical Sciences, Tehran, Iran; c Department of Radiation Oncology, University of Texas Southwestern Medical Center, Dallas, TX, USA; d Department of Radiation Oncology, Mayo Clinic, Rochester, MN, USA

**Keywords:** Metastatic rectal cancer, Biomarkers, Organ preservation, Quality of life, Radiotherapy

## Abstract

About 20% of patients with rectal cancer present with metastatic rectal cancer (MRC). The 5-year relative survival for patients with MRC is about 15%. With emerging systemic therapies, including more effective chemotherapy regimens, immunotherapy, targeted therapies, and radiotherapy as a local option, more patients with advanced rectal cancer are achieving complete clinical response in the primary tumor. Consequently, non-operative management (NOM) through a “watch and wait approach” is becoming increasingly desired. Additionally, improved biomarkers, such as circulating tumor DNA (ctDNA), can improve accuracy in predicting responses to neoadjuvant chemoradiotherapy and monitoring recurrence after achieving complete clinical response. In this narrative review, we examine the current evidence on the role of radiotherapy in the management of MRC, with a focus on NOM and organ preservation strategies. Notably, although several investigations have evaluated various aspects of the NOM approach, there is still limited data regarding patient satisfaction and quality of life. The improved quality of life may be a key factor driving patient preference for NOM, and we also emphasize the data on the impact of NOM on patient’s quality of life.

## Highlights

• Radiotherapy offers an opportunity for non-operative management and organ preservation in metastatic rectal cancer.
• Radiotherapy has the potential to be integrated into the management of patients with metastatic rectal cancer as a curative or palliative option.
• Optimized biomarkers can identify patients likely to benefit from radiotherapy in organ preservation.

## Graphical abstract

The pivotal role of radiotherapy in integrating non-operative management in the treatment of resectable and unresectable metastatic rectal cancer. cCR: clinical complete response; CRT: chemo-radiotherapy; NOM: non-operative approach.

**Figure:**
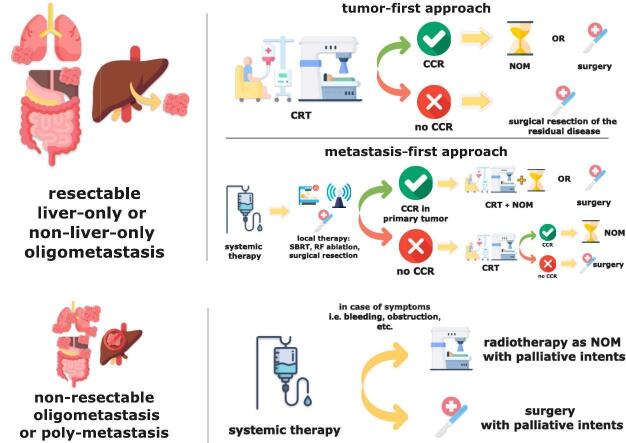

## Introduction

Colorectal cancer (CRC) remains the third most commonly diagnosed cancer and the second leading cause of cancer-related deaths worldwide, with rectal cancer accounting for approximately one-third of all CRC cases [1]. At the time of diagnosis, around 20 % of patients with rectal cancer present with synchronous distant metastases [2]. The 5-year relative survival for patients with metastatic rectal cancer (MRC) is reported to be about 15 % [2]. Nevertheless, the site of metastasis, number, and tumor staging affect survival with some patients able to achieve a long-term disease control [3]. The most common sites of metastasis are the liver and lung [3]. Based on the latest version of the AJCC staging manual, metastatic or stage IV CRC is categorized based on its extent of metastasis: IVa for metastasis to one site or organ, IVb for metastasis to multiple sites or organs, and IVc for metastasis with peritoneal involvement [3].

Metastatic disease is categorized as resectable or unresectable. In addition to the performance status of the patient and availability of the surgical skills and devices, the resectability of metastatic foci takes into account the number and location of metastatic lesions, the health status of the affected organ and the expected function of that organ after resection [[3], [4], [5]]. Resectable metastases are usually located in a few sites in the liver or lung and *not* in the peritoneum, distant lymph nodes, bone, or the brain. After a good response to systemic therapy, some patients with initially unresectable MRC in the liver or lung become converted to resectable disease [[6], [7], [8], [9]].

Systemic therapeutic regimens form the pillar of MRC treatment. Chemotherapy, the key component of systemic therapy for MRC, includes regimens based on fluoropyrimidine (5-FU or Capecitabine) and can be combined with either oxaliplatin (FOLFOX or CAPEOX) or irinotecan (FOLFIRI or CAPIRI) [[10], [11], [12]]. In addition to historical chemotherapeutic agents, the ability to analyze individual tumors for molecular biomarkers of response or resistance, such as microsatellite instability (MSI) or mismatch repair (MMR), KRAS, NRAS, BRAF, and HER-2 genes, has been helpful in personalization of systemic therapies [[13], [14], [15]]. For instance, immunotherapies like PD-1 and CTLA-4 inhibitors can target tumors with MMR deficiency (dMMR) or high MSI (MSI-H), with enhanced survival outcomes [[16], [17], [18]]. Recent studies in rectal cancer have shown promising results for novel systemic therapies,particularly in tumors characterized by dMMR or MSI-H. For instance, neoadjuvant PD-1 blockade with Dostarlimab resulted in a 100 % clinical complete response in patients with locally advanced dMMR rectal cancer. In this small cohort, none of the patients required surgery or radiotherapy, and no disease progression or grade 3 or higher adverse events were observed during follow-up [19], providing proof-of-concept that both surgery and radiotherapy might be unnecessary in selected patients with dMMR rectal cancer. Moreover, as a consequence of the enhanced efficacy of novel therapies, better response has been observed in MRC, in both metastatic sites and in the primary tumor which is becoming more predictable by novel techniques like circulating tumor cells or cell-free DNA [20].

For non-metastatic locally advanced rectal cancer (LARC) patients, neoadjuvant chemoradiotherapy (NCRT) followed by total mesorectal excision (TME) has been historically the standard of care. Total neoadjuvant therapy (TNT) is the new standard of care for LARC [21]. Due to the improved rate of complete clinical response to neoadjuvant therapies by implementing TNT, the excellent outcomes of these patients, and also the quality-of-life issues with TME, there has been a growing interest in non-operative management (NOM) in recent years, as a strategy for organ preservation [22]. On the other hand, in MRC, with emerging systemic therapeutic strategies, such as more effective regimens of chemotherapy, immunotherapy, and targeted therapies, and also radiotherapy as a local non-operative option, more patients are reaching a *complete clinical response* [6,8,[23], [24], [25]], hence, NOM under a “watch-and-wait” or an “active surveillance” approach seems more feasible than ever. The prognoses for different subclassifications of MRC might differ; however, both groups of patients with either resectable or ablatable oligometastatic disease and non-targetable poly-metastatic disease could benefit from an organ-preserving and NOM of the primary tumor, albeit in different settings. For the former group, organ preservation could serve as a curative approach, while for the latter, it could act as a palliative measure, both with the potential intent of sparing patients’ functionality and quality of life.

In this article, we review the evidence focusing on the role of radiotherapy in the management of MRC, with an emphasis on its potential to enable NOM and organ preservation in both resectable and unresectable disease. We aim to explore how radiotherapy can contribute to organ preservation strategies in curative-intent cases as well as provide symptom control and improved quality of life in palliative settings. While systemic therapies and metastasis-directed treatments are briefly discussed, they are addressed in relation to their impact on the feasibility of NOM and organ preservation of the primary tumor.

## Literature review methodology

This narrative review is based on a comprehensive literature search conducted in PubMed and Google Scholar, covering publications from 2000 to 2024. Additional sources included clinical guidelines from major radiation oncology societies, such as the European Society for Radiotherapy and Oncology (ESTRO), and American Society for Radiation Oncology (ASTRO).

The following search terms and combinations were used: “metastatic rectal cancer,” “radiotherapy,” “non-operative management,” “organ preservation,” “oligometastatic disease,” “systemic therapy,” “chemoradiotherapy,” “watch and wait,” and “quality of life.” Both Medical Subject Headings (MeSH) and free-text terms were employed where applicable.

We included original studies, clinical trials, *meta*-analyses, systematic reviews, consensus guidelines, and expert opinions published in English. The selection of articles was based on their relevance to the main topic, with a particular focus on non-operative management and organ preservation strategies. The references of the included articles were also manually screened to identify additional relevant studies.

### Concerns and rationale for proposing organ preservation in MRC patients

Compared to the operative approaches, NOM might provide patients with spared functional outcomes, and subsequently a relatively improved QoL in the long term. The improved QoL could be considered as one of the main reasons patients might be willing to undergo an NOM. This potential improvement in quality of life is particularly significant given the detrimental effects commonly associated with prolonged systemic therapies in metastatic patients, including chronic fatigue, gastrointestinal toxicity, neuropathy, and the cumulative physical and psychological burden of continuous treatment.

Although investigations have evaluated various aspects of the NOM approach, there are still limited data regarding patients’ satisfaction and QoL [26]. In comparison to other types of cancer, such as breast and prostate cancer, where there are more available treatment choices, data on MRC patients’ preferences are also scarcer. Overall, the decision-making process regarding treatment options is considerably complex, with several factors playing a crucial role, including cure rates, thoughts, and beliefs, fear of disease progression, fear of side effects, sexual function, QoL, and the desire for physical removal of the tumor by surgery rather than radiotherapy [27].

On the other hand, Standardized outcome measures are essential to evaluate and compare NOM strategies and organ preservation outcomes. For instance, the international consensus recommendations by Fokas et al. provide key definitions and benchmarks for assessing clinical complete response, local regrowth, salvage surgery, and functional outcomes in this context [28]. Incorporating these standardized metrics ensures consistency in reporting and facilitates comparison across different studies.

Previous studies suggested that the majority of patients with breast and prostate cancer believe that the only permanent curative option is surgery [29,30]. It seems that such beliefs can influence the decision of patients with MRC when confronted with an NOM treatment option. From the patients’ perspectives, it is found that a higher willingness to take risks influences the decision-making process. Research has shown that a more toxic radiotherapy protocol, as part of the NOM trial, was the most critical factor preventing patients from accepting it. This reluctance appears to stem from misperceptions about radiotherapy [31], [28], with the majority of patients viewing requiring radiotherapy as terrible news, brutal, and inherently damaging [32], [29]. Regardless of several recent advancements in reducing radiotherapy side effects, these concerns remain significant for 80 % of patients [33]. Moreover, recent events such as the media portrayal of the PROSPECT trial [34] results have further contributed to public anxiety regarding radiotherapy. Following the publication of this trial, there was widespread media coverage that, according to ESTRO, failed to present a balanced view, leading to public misunderstanding and concern. In response, ESTRO issued a statement urging responsible communication that accurately reflects scientific evidence [35]. Additionally, Wawrzuta et al. demonstrated a rise in negative portrayals of radiation oncology in media reporting, emphasizing the need for improved public education and balanced media narratives, in addition to radiation oncologists actively addressing these misrepresentations and their potential impact on patients’ decision-making regarding treatment [36].

Concerns regarding the safety of NOM usually include the possibility of synchronous distant spread with local regrowth, resulting in an incurable disease. However, NOM did not compromise long-term cure rates in patients who developed local regrowth and underwent salvage surgery [37]. In a recent series of patients who underwent NOM, the risk of local regrowth with distant failures was reported to be around 2 % [38]. On the other hand, some other studies reported higher risks of local regrowth. For instance, Chadi et al reported a local regrowth rate of 37 % for patients with an initial cT4 tumor, with the rate being associated with the tumor stage [39]. Moreover, the risk of distant metastasis has been demonstrated to be significantly higher in patients with local regrowth compared to those without it [40]. This risk has been also higher in patients with local regrowth under the WW approach than in patients undergoing upfront surgery after NCRT [41]. These findings underscore the complexity of determining the safety of NOM, as the risk of local regrowth and distant metastases appears to vary based on initial tumor staging and patient selection. While some studies suggest that NOM does not compromise long-term cure rates when salvage surgery is performed, others highlight a significantly higher risk of recurrence, particularly in patients with advanced tumors. This discrepancy emphasizes the need for personalized decision-making, incorporating tumor characteristics, patient preferences, and rigorous follow-up strategies to optimize outcomes while balancing the benefits of organ preservation against the potential oncologic risk. To conclude we should say that although we know that MRC patients might be at higher risk for local re-growth due to the aggressiveness of the primary tumor, we still can propose organ preservation in case of a complete clinical response of primary tumors based on more strict criteria for both response assessments and routine follow-up visits that address these concerns.

That being said, a recent study showed that fear of recurrence was an obstacle to selecting the organ preservation method in only less than 10 % of participants [42]… Fear of surgery, including its long-term impairments such as permanent colostomy can also influence patients’ decision-making [43], [44]. In fact, Kennedy et al. reported that patients are willing to tolerate a considerable local regrowth risk of 20 % and a 20 % decrease in OS to receive an organ-sparing NOM instead of abdominal perineal resection. Interestingly, the accepted increase in local regrowth and decrease in OS by physicians was only 5 % [45]. This study sheds light on the differences in the perspectives and preferences of patients and medical professionals. This also underscores the importance of informing patients on all aspects of the therapeutic options, rather than merely focusing on oncological outcomes.

Functional outcomes after surgery have been studied more extensively than treatments in NOM. Sexual dysfunction has been reported to be as high as 70 % in patients after curative surgery in patients with rectal cancer, with a more substantial effect on women [46]. Regarding male sexual dysfunction, while a history of pelvic irradiation might increase its risk after surgery [47], the risk of erectile dysfunction might be lower in patients receiving radiotherapy under NOM. For instance, Custers et al. reported an erectile dysfunction rate of about 32 % in patients under an NOM approach [48],compared to the reported rate of more than 60 % after surgery [46],. In a more long-term follow-up setting, studies on patients from a randomized clinical trial of pre-operative short-course radiotherapy (SCRT) and total mesorectal excision (TME) vs. TME alone, reported receiving the preoperative radiotherapy to be a risk factor for experiencing major low anterior resection syndrome (LARS) in patients 14 years after the treatment [49]. Moreover, patients who underwent RT + TME reported symptoms associated with bowel dysfunction and also erectile dysfunction more than the patients in the TME group. However, the global health status and overall function between the two treatment groups did not differ, while slightly decreased compared to the normal population [50].

While in comparative studies including surgery versus radiotherapy followed by surgery, the reported QoL is reported to be slightly more diminished in the irradiated patients, with the introduction of the NOM, studies sought to investigate if that would still be the case in the absence of surgery. A study [51] comparing radiotherapy alone with radiotherapy followed by TME suggested that patients in both groups reported impaired QoL, but outcomes for the radiotherapy alone group were substantially better in several functional aspects. In a recent investigation of patients attending surveillance after any of four treatments (RT, local excision (LE), radiotherapy then LE, or LE then RT), the overall QoL scores were similar between the groups at a median of 10 months following treatment, indicating fairly good QoL [52]. In another study comprising patients on an “active surveillance” approach, from whom about 80 % proceeded to need surgery, NOM was associated with less LARS. Besides NOM, female sex and lower BMI at the time of receiving RT, but not SCRT vs LCRT, were associated with better QoL outcomes [47]. Both sexual and bowel function outcomes have been reported to worsen in patients under an active surveillance approach who then undergo surgical resection [48]. Overall, it seems that organ preservation in NOM provides patients with more satisfaction and QoL. However, in case of relapse or local recurrence, when the patients need to switch to an operative approach, the outcomes seem to deteriorate. Moreover, it should be noted that most of these available results come from patients with LARC, and comprehensive assessments on the NOM in patients with MRC, particularly resectable MRC, are yet to be performed.

### Metastasis-directed therapies: enabling organ preservation strategies in metastatic rectal cancer

As mentioned before, stage IV or M1 disease can be stratified into IVa, b, or c, based on the tumor spread into one distant metastatic site, two or more sites, or the peritoneum, respectively. Unlike many other stage IV cancers, surgical removal of MRC can substantially extend survival, and in certain instances, resection of liver and lung metastases can lead to curative outcomes [6], [7], [8]. Some studies could even demonstrate a survival benefit with surgical intervention for peritoneal metastases compared to palliative care [53], [54]. Therefore, a refined classification of the MRC might contribute to selecting the right therapeutic approach for the patients, underscoring different prognoses for different subclassifications of MRC, i.e. 1) resectable liver-only, 2) unresectable, liver-only, 3) non-liver only oligo-, and 4) poly-metastasis.

Controlling the metastasis by improvement of metastasis-directed therapies renders NOM feasible. Effective control of metastatic disease through metastasis-directed therapies not only improves systemic disease outcomes but also creates a clinical context in which successful organ preservation of the primary rectal tumor becomes possible, particularly in patients with resectable oligometastatic disease.

While the role of local removal of the metastasis in resectable liver-only MRC is more established, the impact of metastasis-directed therapies in other groups is being studied. In the case of MRC with multiple metastatic sites (poly-metastasis), the results of the ORCHESTRA trial demonstrated no additional benefit in patients’ survival by maximal cytoreduction, i.e. local tumor debulking by either surgical resection, radiotherapy, or thermal ablative therapy [55]. ERASUR trial is also exploring the survival outcomes in patients with non-liver-only oligometastatic disease, i.e. four or fewer metastatic sites, that are not only in the liver, going through total ablation of known metastases [56]. The trial is accruing.

Surgical resection has been established as the standard of care for colorectal liver metastases (CRLMs) in patients with resectable liver-only disease. However, we should note that about 80 % of CRLM are not candidates for surgery [57]. However, factors like two or more liver metastases, size of the largest metastasis foci > 6 cm, and major hepatectomy were among the factors associated with worse survival in patients undergoing hepatic metastasectomy [58]. Other therapies targeting metastases, include thermal ablation (such as radiofrequency ablation (RFA) and microwave ablation (MWA)), stereotactic body radiation therapy (SBRT), *trans*-arterial chemoembolization (TACE), and radioembolization (TARE). These approaches can be utilized for resectable liver metastases and also aim to improve resectability and provide curative treatment options for patients with unresectable, liver-only, or non-liver-only oligometastatic disease, increasing their chance to continue their treatment under NOM. When used in conjunction with systemic therapy, ablative therapies can improve disease-free survival (DFS) and OS compared to chemotherapy alone [59], [60]. These methods can also be safely integrated with surgery in cases of CRLMs, offering recurrence-free survival (RFS) and OS rates comparable to surgery alone [61]. In fact, the COLLISION trial demonstrated thermal ablation to be non-inferior to surgical resection in terms of progression-free survival (PFS) or OS, improving safety and local control for patients with resectable liver-only MRC [62]. Local ablative treatments can be utilized either independently or alongside surgery for resectable pulmonary metastases, as well [63]. Thermal ablation methods even showed improved survival outcomes when surgical resection is not feasible [64], [65]. MWA and SBRT appeared equally effective for tumors of intermediate size, 3–5 cm, in achieving local tumor control [66], however, for smaller tumors, SBRT may be considered for centrally located lesions that are not suitable for thermal ablation [66].

In this context, although surgery is still the standard of care for resectable liver or lung metastases of the rectal cancer, there is a potential window of opportunity for SBRT as an alternative to surgery. SBRT compared to other local therapies and surgery could have high local control, less anatomical limitations, non-invasiveness, minimal or no interruptions with systemic anti-cancer therapies, ability to target multiple lesions at the same time and to repeat the procedure over time, and ability to target larger lesions [67], [68]. Until then, the choice of best metastasis-directed therapy is recommended to be made by a multidisciplinary team.

## Primary organ preservation in resectable metastatic patients with curative intent

Resectable MRC can have a spectrum of prognoses from very good as a non-metastatic disease to very poor as an aggressive, unresectable disease [15], [69], [70], [71], [72]. Resectable MRC can be managed in two major paths. One path starts with surgical resection of the metastatic foci to avoid unresectability over time and continues with a course of systemic therapy and radiotherapy followed by surgery for the primary tumor. Another path starts with an abbreviated course of systemic therapy, usually with doublet chemotherapy alone, to observe the biologic nature of the tumor and, in case of partial response or stable disease, continues with surgical resection of the metastatic disease and local treatment of the pelvic disease [15], [70], [73]. Aside from which path is chosen, there are two main approaches when addressing surgery for metastases and pelvic disease. For example, in liver-confined MRC we can name liver-first (staged resection) and rectum-first approaches (synchronous resection) [15], [70]. Both approaches are popular and have their pros and cons.

When focusing on the management of pelvic disease after addressing the metastases by surgical resection, ablation or SBRT, one can consider a similar approach to non-metastatic locally-advance RC despite the fact that these patients have not been included in any “Watch and Wait” trials so far. Indeed, when the metastatic foci are removed, the chance of cure might be as much as a locally advanced disease. We should bear in mind that the outcomes of such patients are usually determined by the systemic disease and not local disease. Accordingly, in cases with a clinically complete response after radiotherapy, we can opt for the watch-and-wait approach with more emphasis on the detection of systemic disease. In such cases, there are no perceived contraindications for recommending NOM like an initially non-metastatic locally advanced disease.

### Primary organ preservation in unresectable metastatic disease with palliative means

Advancements in targeted therapies offer hope for improving outcomes in unresectable diseases. Identifying potentially effective targeted therapies by detecting somatic variants of the tumoral genes could improve the OS in tumors with specific genomic profiles. For instance, for metastatic KRAS/NRAS/BRAF wild-type tumors, a combination of chemotherapy and monoclonal antibodies to the epithelial growth factor receptor (EGFR), i.e. cetuximab and panitumumab, can reportedly increase median survival by 2 to 4 months compared to chemotherapy alone [74]. Nowadays, a patient with unresectable metastatic disease may live up to 38 months maximum.

In the subgroup of patients with unresectable disease, there may be indications for local therapies. As the metastases define the course of the disease and patients’ outcomes, the major efforts should be on a continuum of effective systemic therapy. Removing the primary tumor in asymptomatic patients has been studied without any documented benefits [15], [75]. In several reviews and randomized controlled trials, researchers did not find any survival benefit from primary tumor resection in the setting of unresectable metastatic CRC. It appears that the mortality in these patients is mainly due to their systemic disease rather than complications related to the primary tumor [76]. However, in these patients, due to local symptoms that are resistant to systemic therapy, including bleeding, obstruction, and pain, there may be indications for local therapy such as endovascular procedures (e.g., embolization, coiling, stents), which can provide quick and effective palliation [77], [78], [79]. Surgery and radiotherapy are both viable options in this regard. Surgery relieves the symptoms faster at the cost of withholding systemic therapy for a couple of weeks to allow for wound healing and anastomosis maintenance. In the case of receiving bevacizumab, this time of systemic therapy withdrawal could be longer [15], [80], [81].

While radiotherapy alleviates symptoms more slowly than surgery, it does not require the suspension of systemic therapy [82]. Radiotherapy has been reported to be effective in palliating symptoms such as pain, bleeding, and mass effects, without any substantial adverse effects [75]. In a phase II clinical trial, SCRT was able to reduce or stop pain and bleeding in 87.5 and 100 % of the enrolled patients, respectively. SCRT could also avoid the need for colostomy in patients with unresectable MRC, as in 3 years, the rate of colostomy-free survival was 47.6 %, which could be promising [83]. Thus, in unresectable MRC patients, radiotherapy can exert its role as an effective organ-preserving palliative treatment without posing the patients with the risks of surgery and providing them with a better QoL. Table 1 summarizes key clinical pathways and the placement of RT and implementation of NOM in both resectable and unresectable MRC clinical scenarios (Table 1).

**Table 1:** Potential role of radiotherapy in primary tumor management and non-operative management (NOM) strategies in metastatic rectal cancer.

Setting	Strategy	Key Steps	Role of Radiotherapy / NOM	Key Considerations
Resectable MRC	Surgery-first	Metastases resection → systemic therapy → primary RT ± surgery	RT may lead to cCR in primary tumor; NOM considered after metastasis control	Comparable prognosis to non-metastatic cases if systemic disease controlled
	Systemic therapy-first	Systemic therapy → metastasis resection → local treatment	RT post-metastasis resection; NOM feasible in cCR	Selection of indolent tumors; systemic control is key
Unresectable MRC	Palliative RT	Systemic therapy ± targeted agents; local palliation if needed	RT relieves symptoms without interrupting therapy	Could improve QoL without posing the risks of surgery

MRC: metastatic rectal cancer; QoL: quality of life; RT: radiotherapy.

### The choice between SCRT and LCCRT for primary tumor in MRC

In de-novo resectable cases, as the NCCN points out, both SCRT and long-course chemoradiotherapy (LCCRT) are viable options for resectable MRC based on the risk factors for local recurrence in the primary tumor [15]. Thus, The choice of radiotherapy regimen (SCRT vs. LCCRT) has implications for organ preservation strategies and NOM feasibility in patients with MRC.

One notion is that as these patients benefit long survivals like those without metastases, we might follow the same decision-making in M0 patients. First, we assume that surgery is a part of the local treatment of the primary tumor. Taking the long-term results of the RAPIDO trial into account, we can say that one should be cautious when opting for SCRT due to the higher risk of local recurrence [84]. There is a strong relationship between tumor depth of invasion, or T stage, and the chance of distant metastases. As Ptok et al. clearly stated the chance of distant metastases increased steadily from 3.5 % in pT2 to 47 % in T4 cases [85]. Thus, we should bear in mind that we usually face a bulky, locally advanced primary tumor in the setting of MRC that is also at higher risk for local recurrence [86], which may not be an ideal candidate for SCRT in the first place. On the other hand, some believe that as the systemic disease determines the final outcome of the de novo MRC, every effort should be made to continue the courses of systemic therapy as soon as possible. In this regard, the higher local recurrence rate of 10 % vs. 6 % in the RAPIDO may be negligible. So, as the competing risks of distant recurrence is so higher than M0 patients, SCRT becomes their schedule of choice, giving all the radiotherapy fractions over a week with or without a concomitant boost to extra-mesorectal lymph nodes [87]. In this point of view, LCCRT remains for those primary tumors which need a dramatic response to become resectable as the chance of R1 resection is so high. In these scenarios, LCCRT is done following a sufficient course of systemic therapy. There is no robust evidence to suggest one approach over another but it seems logical to consider the risk factors of local recurrence into account when deciding between SCRT and LCCRT [88].

In fact, when a patient is a candidate for local therapy, they should be considered as an M0 patient who might have long-term OS, although keeping in mind that the competing risks of distant failures is so high. Second, if we aim to have a NOM approach in a shared decision-making with the patient, as the bulk of evidence suggests, we should use the LCCRT schedule ignoring the risk of distant regrowth over the course of RT. Knowing the fact that based on the OPRA trial starting the local treatment with CRT followed by consolidation chemotherapy is superior to induction chemotherapy followed by CRT in terms of TME-free survival and cCR [90], [91], due to the inherent concerns of distant failure in MRC, we are of no choice other than offering a consolidation CRT. However, the complete clinical responders may have a better OS afterward [92].

In an unresectable synchronous MRC, radiotherapy is usually a treatment with palliative intent. SCRT can provide faster and equally effective palliation compared to LCCRT [93]. In occasional cases with metastases that are converted to resectable after first-line systemic therapy, SCRT and LCCRT are both considerable. However, due to the increased concern of regrowth of metastatic foci during radiotherapy in comparison to initially resectable MRC, SCRT is usually preferred to continue the systemic therapy sooner as a bridge to pelvic surgery when the metastatic foci are still resectable. If the metastatic foci are completely resolved by the induction systemic therapy, SCRT might still be the preferred option for these patients as the competing risk of distant regrowth is highest in this group of patients compared to initially resectable M1 patients. Due to the high chance of systemic spread and the potential superiority of SCRT in this scenario, we should be aware of the high chance of re-growth after a clinical complete response (cCR) in the primary tumor [89].

### Emerging radiotherapy strategies to optimize non-operative management in metastatic rectal cancer

Following the same notion that a subset of resectable MRC patients can enjoy long survival rates close to M0 patients, they are eligible to undergo radiotherapy techniques that can boost their chances of having a cCR. Again, none of the main trials in this regard have recruited MRC patients; thus we can follow the same rules as M0 patients. There are a number of ways to enhance the chance of achieving a cCR, with most of them tested in small, non-randomized clinical trials except for the electronic brachytherapy device, the Papillion method, in the OPERA trial [94]. Other options include delivering a simultaneous integrated boost (SIB) by volumetric arc therapy (VMAT) [95], [96], or giving a boost by magnetic resonance linear accelerator (MRLinac) [97]. These options should be discussed with patients who wish to preserve their rectum considering the mandate of available machines and the skilled radiation oncologists and staff.

### Role of predictive and prognostic factors in guiding toward organ preservation

Good patient selection is crucial when discussing OP. By the emergence of metastasis, we already know that the patient is at high risk of distant failure. So, eventually, the risk factors for distant failures, such as N2 or EMVI or a high CEA, fade in decision-making. Hence, pelvic failure risk factors such as T4, extra-mesorectal node, and a low-lying tumor become more critical when discussing the goals of care with patients and opting for the radiotherapy schedule and sequence and the technique to achieve the maximal clinical response [88]. Table 1 summarizes the main findings of the studies that investigated the clinical implementation and clinical value of ctDNA in the preoperative setting.

In addition to conventional prognostic factors, such as tissue biomarkers and ctDNA, advanced imaging analysis techniques, including radiomics, are emerging as promising tools for assessing treatment response in rectal cancer. For instance, radiomic features extracted from CT scans or MRI have been investigated for their ability to predict pathological complete response (pCR) after NCRT in patients with LARC, often demonstrating superior predictive value compared to conventional clinical and radiological methods—even prior to the initiation of treatment [98]. Furthermore, some studies have developed integrated predictive models that combine radiomics with features extracted from biopsy histopathological images or pathomics, enhancing the accuracy of treatment response prediction [99], [100]. Artificial intelligence (AI) and machine learning algorithms using radiomics and pathomics could enable more precise patient selection for organ preservation and NOM strategies in rectal cancer; however, further prospective studies might be needed to demonstrate their practicality and efficacy in real-world clinical settings.

## Future directions and conclusion

Managing MRC is complex due to the variability in tumor extent and molecular characteristics. Advances in systemic therapy, a deeper understanding of tumor biology, and the significance of molecular profiling have led to prolonged survival for more patients with MRC. As we witness better survival outcomes in MRC, preserving and improving patients' quality of life is becoming more critical. While our review highlights NOM and organ preservation strategies, it is important to acknowledge that, in certain cases, proceeding directly to surgical resection following systemic therapy—without radiotherapy—may be a valid approach. This is particularly relevant when resection can be achieved without compromising function, as demonstrated by recent data from the PROSPECT trial [31]. It is worth noting that PROSPECT patients were at relatively low risk for locoregional recurrence compared to the other TNT approach-based trials. The presumed assumption that metastatic patients usually have more advanced primary tumors makes PROSPECT a not-so-valid option to achieve maximum local control currently. However, by a good patient selection, organ preservation especially without surgery can yield better QoL and reasonable control of the primary tumor.

Future research directions include optimizing treatment regimens to improve oncological outcomes while minimizing treatment-related morbidity. This includes investigating the role of novel systemic therapies, such as targeted agents and immunotherapies, in conjunction with radiotherapy and potentially less radical surgery. Additionally, the development of better risk stratification tools will be crucial for selecting patients who are most likely to benefit from organ preservation strategies. Finally, further research is needed to identify biomarkers that can predict response to treatment and identify patients at risk for recurrence after organ preservation. This will allow for more personalized treatment approaches and ultimately improve patient outcomes.

## Funding

Not applicable.

## Declaration of competing interest

The authors declare that they have no known competing financial interests or personal relationships that could have appeared to influence the work reported in this paper.
